# Polarimetric analysis of the Alzheimer’s pathology in excised mouse brain tissue

**DOI:** 10.1117/1.JBO.31.1.015002

**Published:** 2026-01-28

**Authors:** Gaurav Sharma, Jens Pahnke, Bernhard Roth

**Affiliations:** a Leibniz University Hannover, Hannover Centre for Optical Technologies, Hannover, Germany; b Oslo University Hospital, University of Oslo and Section of Neuropathology Research, Translational Neurodegeneration Research and Neuropathology Lab, Department of Clinical Medicine, Department of Pathology, Medical Faculty, Clinics for Laboratory Medicine, Oslo, Norway; c University of Lübeck, University Medical Center Schleswig-Holstein, Institute of Nutritional Medicine, Lübeck, Germany; d University of Latvia, Department of Neuromedicine and Neuroscience, Faculty of Medicine and Life Sciences, Riga, Latvia; e Tel Aviv University, School of Neurobiology, Biochemistry and Biophysics, Department of Neurobiology, The Georg S. Wise Faculty of Life Sciences, Tel Aviv, Israel; f Cluster of Excellence PhoenixD, Hannover, Germany

**Keywords:** Alzheimer’s disease, Mueller matrix polarimetry, brain tissue, polarized light

## Abstract

**Significance:**

Given the rapidly ageing global population and the projected rise in dementia cases, research into Alzheimer’s disease (AD) has become an urgent scientific and medical priority. Continued investigation into the molecular mechanisms and early detection of AD is therefore essential to mitigate its growing personal and societal impact. Most current AD therapies in advanced phases of development target amyloid β-peptide (Aβ) production, aggregation, or accumulation.

**Aim:**

Mueller Matrix Polarimetry (MMP) has evolved into a prominent research subject, with a focus on identifying microstructural changes in biotissues. This is done by investigating light properties, which is especially useful in the early detection of brain cell degradation, among others. We set up and employed experimental MMP at three illuminating laser wavelengths (i.e., 445, 532, and 632 nm).

**Approach:**

We investigated the application of MMP to mouse brain tissue containing Aβ plaques. The investigated samples on glass slides consisted of paraffin-embedded brain and paraffin-embedded tissue slices. The tissues were taken at various stages of ageing, i.e., 75, 100, 125, 150, 175, 200, and 225 days. Paraffin tissue blocks were used as an additional sample set for comparison.

**Results:**

We performed a comparative analysis based on the Mueller matrix elements for each age category and highlighted the importance of certain elements, e.g., $m_{44}$, for further analysis. We also compared the trends of decomposition parameters and could correlate them with the ageing. Contrary to previous studies, we also report on retardation, diattenuation, and polarizance changes for later AD changes.

**Conclusions:**

From the higher-order statistics, we concluded that the mean and standard deviation remained constant across the ages. Skewness values were positive and increased as the age progressed, whereas kurtosis decreased with age. The large available dataset opens the possibility of implementing machine learning methods to assist clinical diagnosis in the future.

## 1 Introduction

Alzheimer’s disease (AD) is a leading cause of dementia, affecting ∼50 million people worldwide, with projections estimating that this number could reach 152 million by 2050. Statistically, AD affects ∼6.5 million Americans aged 65 and older, with estimates indicating that this number could rise to 13 million by 2050. AD contributes to significant morbidity and mortality, with an estimated one in three seniors dying with Alzheimer’s or another dementia.^1^^,^^2^ Under the current trend of increasing longevity of the population, this imposes significant burdens on our society, health care, and economy. Consequently, understanding the progression and impact of AD is crucial for developing effective diagnostic and therapeutic strategies.

AD is characterized by progressive cognitive decline, primarily due to the accumulation of amyloid-β plaques (Aβ) and tau tangles in the brain. Aβ plaques are extracellular deposits primarily composed of aggregated Aβ peptides. The amyloid hypothesis posits that the accumulation of Aβ peptides initiates a cascade leading to neuronal dysfunction and cell death. These plaques are a hallmark of AD pathology and are implicated in neurodegeneration.^3^ Imaging techniques provide structural and functional insights into brain changes associated with AD. Structural imaging techniques such as magnetic resonance imaging (MRI) detect brain atrophy, especially in the hippocampus and medial temporal lobe. Computed tomography (CT) is useful for ruling out other causes of cognitive decline, such as strokes or tumours.^4^ Functional imaging includes fluorodeoxyglucose positron emission tomography (FDG-PET), which assesses glucose metabolism in the brain, being reduced in AD-affected regions, and Aβ-PET imaging, which visualizes amyloid-beta deposits in the brain using tracer chemicals.^5^

Optical methods have been used for AD diagnosis already. Optical coherence tomography has been successful in assessing retinal abnormalities *in vivo* and has indeed provided multiple parameters that allowed for the distinction between normal aged individuals and patients with neurodegenerative diseases. It relies on the earliest detectable disease-specific signs, Aβ plaques. The retina of AD patients undergoes substantial ganglion cell degeneration, thinning of the retinal nerve fibre layer, and loss of axonal projections in the optic nerve, among other abnormalities.^6^ Another work demonstrated a wide-field, quantitative, and label-free imaging approach for mouse brain tissue slices at sub-micrometer resolution, using holographic microscopy combined with an automated scanning system. By applying a modified scattering phase theorem to the measured light field images, the authors extracted quantitative scattering coefficients and anisotropy values, which provide insight into the structural organisation of brain tissue. As a proof of concept, it was shown that these scattering-based parameters can effectively reveal and quantify structural alterations in the brains of mice affected by AD.^7^

MM imaging can provide rich microstructural and optical information of tissues for diagnostic purposes.^8^^–^^11^ It has been applied to differentiate various malignant tissues, such as skin cancer,^12^ cervical cancer,^13^ lung cancer,^14^ or colon cancer.^15^ Mueller matrix polarimetry (MMP) is also able to offer insights into AD diagnostics, being low-cost, simple, and label-free. Previously, similar work was carried out on comparable samples and showed that label-free detection of AD in mouse models is possible.^16^^–^^18^

In this work, we investigate how Mueller matrix polarimetry can provide quantitative insights into the progression of AD. We employ age-labeled APP-tg brain samples prepared as paraffin-embedded blocks and as microtome cut slides, both with and without paraffin. Following the experimental measurements, we present a detailed analysis of the resulting polarimetric data. The interpretation is framed within the context of scattering regimes, which serve as the physical basis for the subsequent statistical evaluation. By analysing higher-order central moments, we identify reliable polarimetric markers of AD progression. In particular, the statistical moments (skewness and kurtosis) of the Mueller matrix elements $m_{22}$, $m_{44}$, and the depolarization parameter exhibit clear age-dependent trends that correlate directly with AD advancement. We also provide a comparison between the paraffin-embedded blocks and the corresponding tissue slides. Building on our previously published research, the work presented here implements laser-based wide-field polarimetry at three discrete wavelengths and exploits the full 4×4 Mueller matrix together with Lu–Chipman decomposition. In addition, we provide an explicit Rayleigh to Mie scattering-regime interpretation of the observed polarimetric signatures.

## 2 Materials and Methods

### 2.1 Mouse Samples

**Fig. 1:**
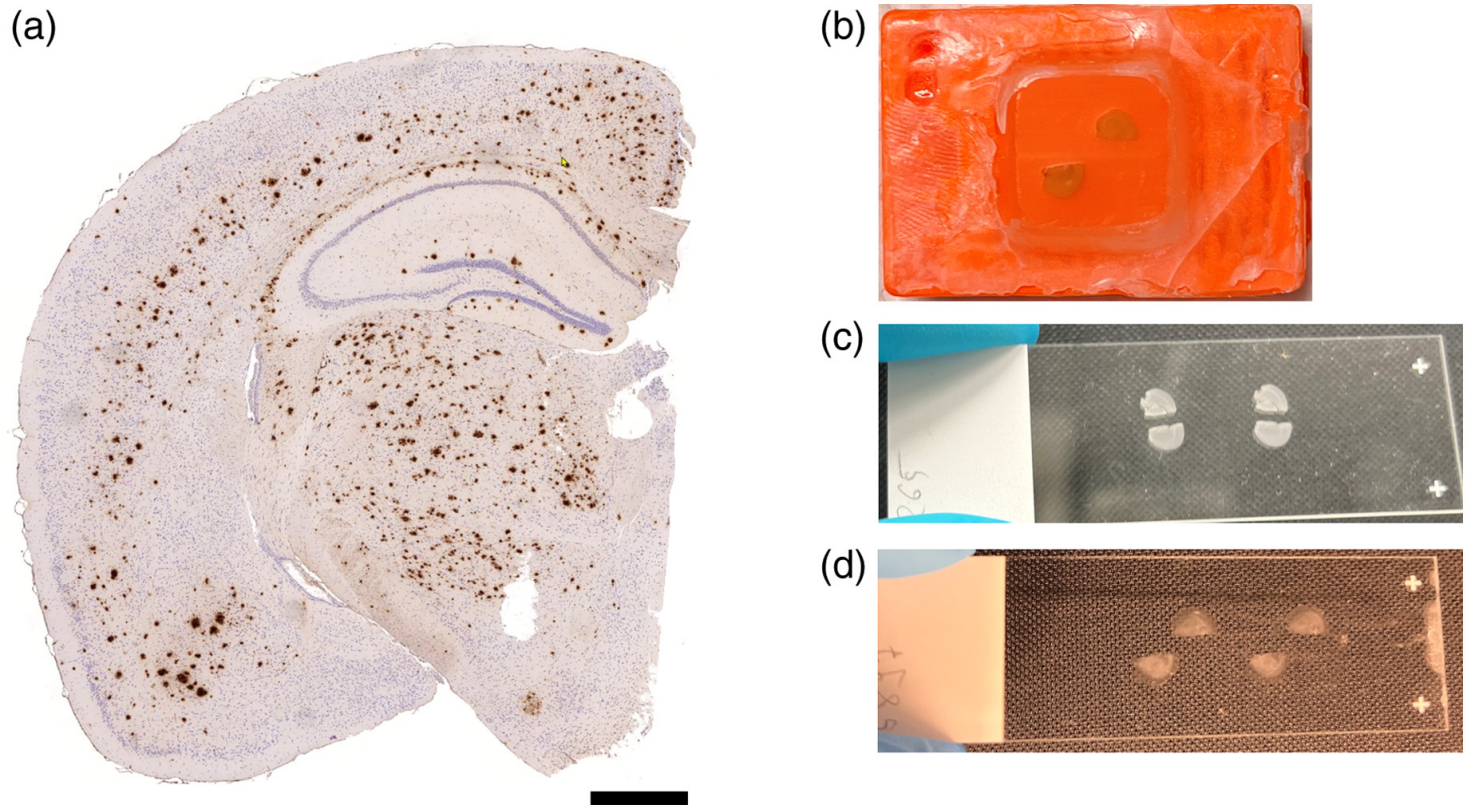
(a) Representative stained microscopy image of a brain section with dark Aβ plaques indicating advanced Alzheimer’s disease (AD). The mouse was 225 days old, scale bar of 400  μm. Sample preparation used in this study is depicted: (b) FFPE block with two brain hemispheres, (c) brain slide without paraffin, and (d) brain slide with paraffin.

In this study, we worked with three types of samples. First, brain samples were sectioned and embedded in formalin-fixed paraffin-embedded (FFPE) blocks. Second, using cryotome sectioning, we prepared two additional sample types: one with paraffin and one without. These sample types are illustrated in Fig. 1. The brain hemispheres were excised from sacrificed APP-transgenic mice (APPPS-21, APPtg) at ages of 75, 100, 125, 150, 175, 200, and 225 days, with a total of 21 samples. The mice are genetically modified to overexpress the human amyloid precursor protein with familial AD mutations and exhibit age-dependent deposition of Aβ plaques in the brain. At an age of 45 days of the animals, the onset of plaque deposition appears. Progression of the plaques is highly correlated with age progression. Microscopy-based imaging has been successful in visualizing Aβ deposition and advancing our understanding of AD pathology.^19^^–^^22^ All procedures related to animal breeding and usage received approval from the Norwegian Food Safety Authority (Mattilsynet).

### 2.2 Mueller Matrix Experimental Setup

MMP delivers information about the polarization-changing properties of the samples under study. This information can be derived from the measurement of the intensity of light which is reflected or transmitted through a sample following illumination at different polarization states. The MM relates to the Stokes formalism used to describe the polarization state of light before and after interaction with the sample.^23^ The Stokes vector $\vec{S}$ is a 1×4 matrix. The element of the Stokes vector, ${\vec{S}}_{1}$, ${\vec{S}}_{2}$, ${\vec{S}}_{3}$, and ${\vec{S}}_{4}$, is associated with total intensity, linear horizontal/ vertical, ±45  deg linear, and right/ left circular polarization, respectively. The Stokes vector of the light field after interaction ${\vec{S}}_{o}$ with a sample can be calculated from the Stokes vector of the incoming light ${\vec{S}}_{i}$ for known MM as follows:

$$(\begin{array}{c} S_{00}\\ S_{o1}\\ S_{o2}\\ S_{o3} \end{array})=[\begin{array}{cccc} M_{11} & M_{12} & M_{13} & M_{14}\\ M_{21} & M_{22} & M_{23} & M_{24}\\ M_{31} & M_{32} & M_{33} & M_{34}\\ M_{41} & M_{42} & M_{43} & M_{44} \end{array}](\begin{array}{c} S_{i0}\\ S_{i1}\\ S_{i2}\\ S_{i3} \end{array}).$$ (1)

Here, the 4×4 MM contains all information about the polarization changing properties of the samples, which can be calculated from measured intensity values I for different polarization states, as shown in Table 1.

**Table 1:** Calculation of the MM elements. The individual elements result from different polarization states of the polarization state generator (PSG) and polarization state analyzer (PSA), the first and second letters on the right side of each matrix element equation. Symbols are as in Eq. (1). All MM images are normalized by the first matrix element M[1,1].

	M[:,1]	M[:,2]	M[:,3]	M[:,4]
M[1,:]	HH + HV + VH + VV	HH + HV − VH − VV	PH + PV − MH − MV	RH + RV − LH − LV
M[2,:]	HH − HV + VH − VV	HH − HV − VH + VV	PH − PV − MH + MV	RH − RV − LH + LV
M[3,:]	HP − HM + VP − VM	HP − HM − VP + VM	PP − PM − MP + MM	RP − RM − LP + LM
M[4,:]	HR − HL + VR − VL	HR − HL − VR + VL	PR − PL − MR + ML	RR − RL − LR + LL

The indices in Table 1 stand for the intensity values stand for the different polarization states of the light (H: horizontal polarization, V: vertical polarization, P: light polarized at an angle of 45 deg, M: light polarized at −45  deg, R: right circular polarization, L: left circular polarization). For measurement of the MM, the change of the Stokes vector after interaction of the incoming light field with the sample is recorded. In total, this typically requires either 16 or 36 different measurements, depending on the polarization states, which need to be generated, i.e., four states (H, V, P, R) or six states (H, V, P, M, R, L), respectively. Details on the formalism can be found in the literature.^24^

**Fig. 2:**
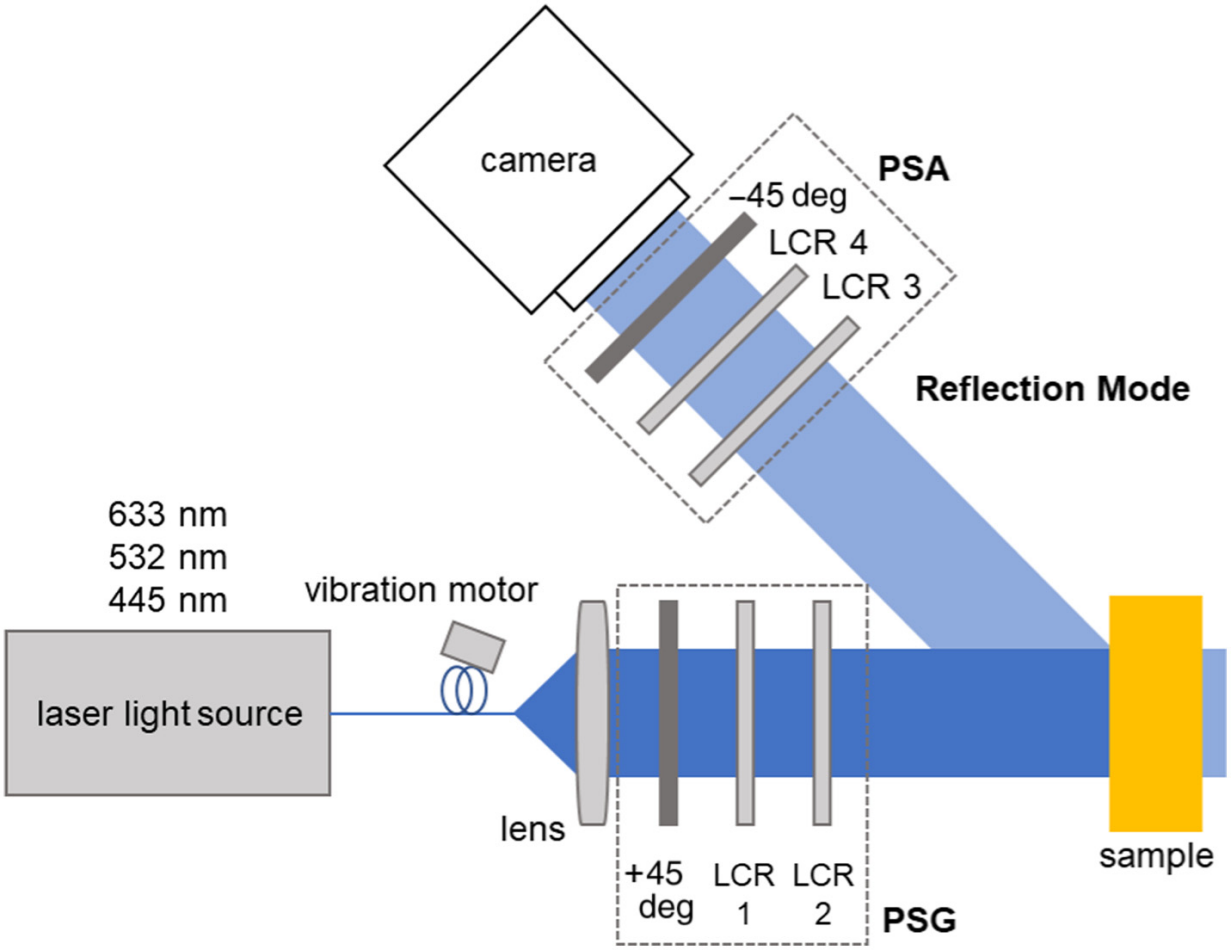
Experimental MM setup. The light from the laser passes through the polarization state generator (PSG), which consists of two liquid crystal retarders (LCR) and a linear polarizer. After the interaction with the sample, the polarization states are analyzed by the polarization state analyzer (PSA), which also consists of LCRs and a linear polarizer. The LCR pairs are placed at different angles with respect to their principal axes.

The wide-field MM measurement system, as shown in Fig. 2, generates the polarization states of the incoming light using a polarization state generator (PSG) and records the outgoing states upon interaction with the samples and subsequently passes through a polarization state analyzer (PSA). The setup has been well described in the published works.^8^^,^^10^^,^^11^ Using a CCD camera (BFS-U3-32S4M-C, FLIR Integrated Imaging Solutions Inc., Richmond, British Columbia, Canada), 2D measurements of the MM of a sample are obtained in one shot. The PSG and PSA components are assembled from fixed polarizers (LPVISE100-A2”, Thorlabs, Newton, New Jersey, USA) and liquid crystal retarders (LCR) (LCC1223T, Thorlabs). By changing the voltage applied to the LCR, all required polarization states can be generated in ∼15  s in total. The system is designed to operate in reflection and transmission mode and uses laser light at different wavelengths, 445 nm (LDMF-Serie VLD-XT 445100, LASOS Lasertechnik GmbH, Jena, Germany), 532 nm (CW532-04-1, Roithner Lasertechnik GmbH, Vienna, Austria), and 633 nm (HeNe, 25-LHR-991-230, CVI Melles Griot GmbH, Bensheim, Germany), for the measurement. From the measured values, Muller matrix element values are calculated, as shown in Table 1.

## 3 Results and Discussion

In this section, we present the polarimetric measurements obtained from the different sample preparation types introduced earlier: slices without paraffin, slices with paraffin, and FFPE blocks. To provide an overview of the raw MM response, we first show representative MM images across three excitation wavelengths (445, 532, and 632 nm). These visualisations highlight the influence of the preparation method on the signal-to-noise ratio (SNR) and the overall contrast of the polarimetric maps.

**Fig. 3:**
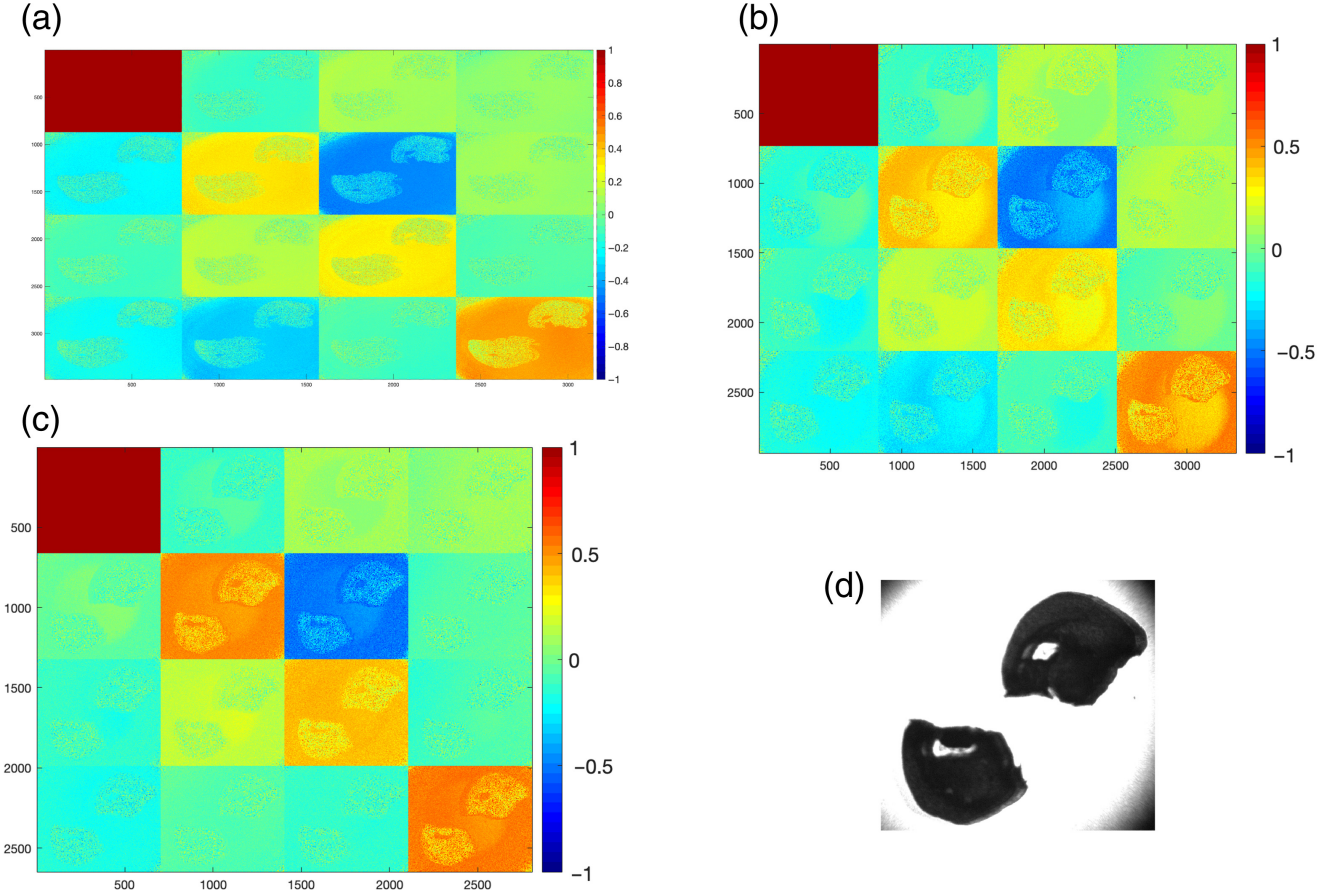
MM of mouse brain slides (prepared without paraffin) for three laser sources, (a) 445 nm, (b) 532 nm, (c) 632 nm, and (d) the photograph of the sample brain under study, age = 225 days.

**Fig. 4:**
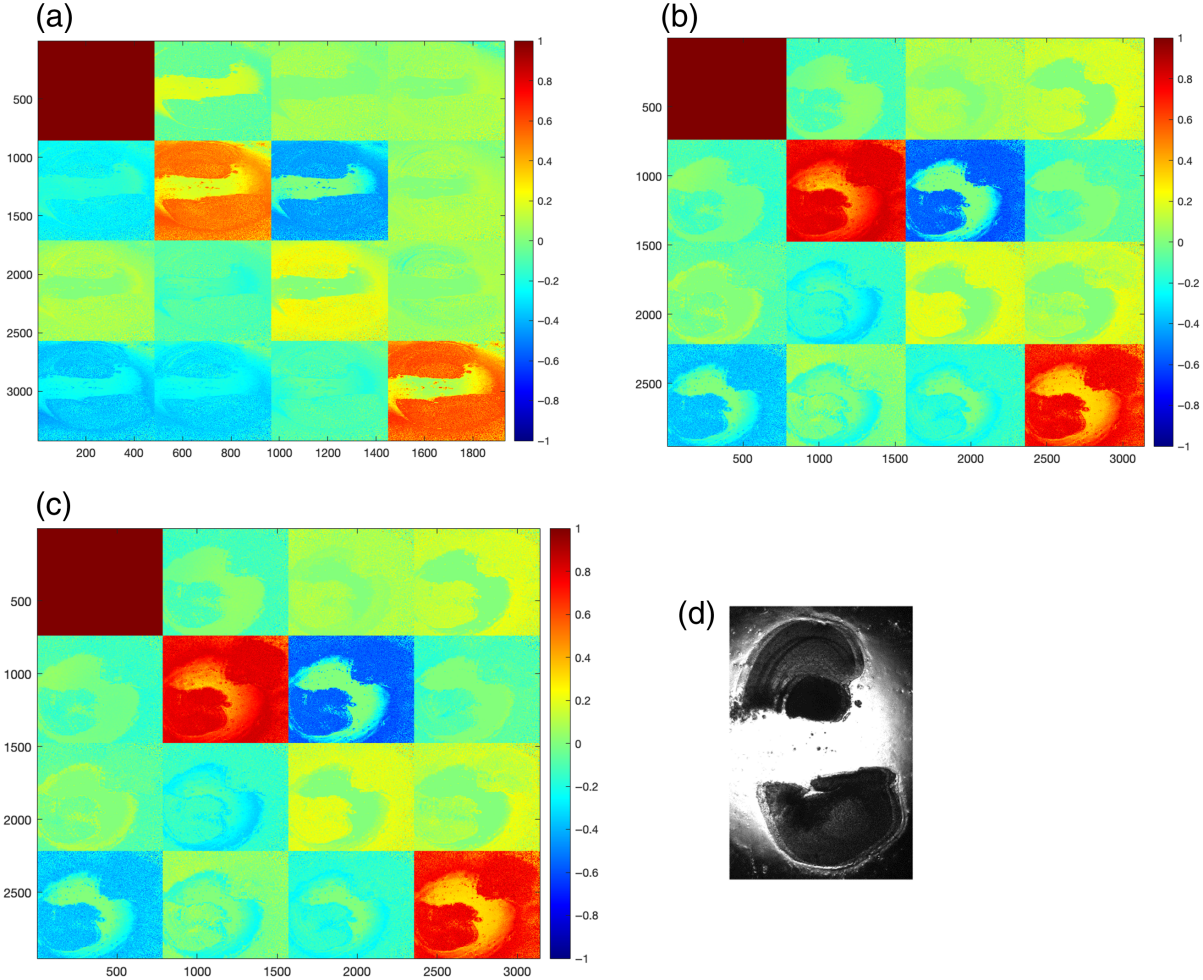
Mueller matrix of mouse brain slides with paraffin for three laser sources, (a) 445 nm, (b) 532 nm, (c) 632 nm, and (d) photograph of the sample brain under study, age = 225 days.

To illustrate the raw polarimetric signatures, Figs. 3 and 4 present representative MM maps of mouse brain slices at an advanced stage of AD (225 days). The maps are shown for three excitation wavelengths (445, 532, and 632 nm), together with the corresponding bright field image of the brain tissue.

In the case of slices prepared without paraffin (Fig. 3), the contrast between brain regions is more pronounced, and the polarization signal is high across the entire matrix. By contrast, paraffin slices (Fig. 4) show visibly reduced contrast and increased background variation, indicating that the embedding medium introduces additional depolarization effects or intensity of the polarization signal.

**Fig. 5:**
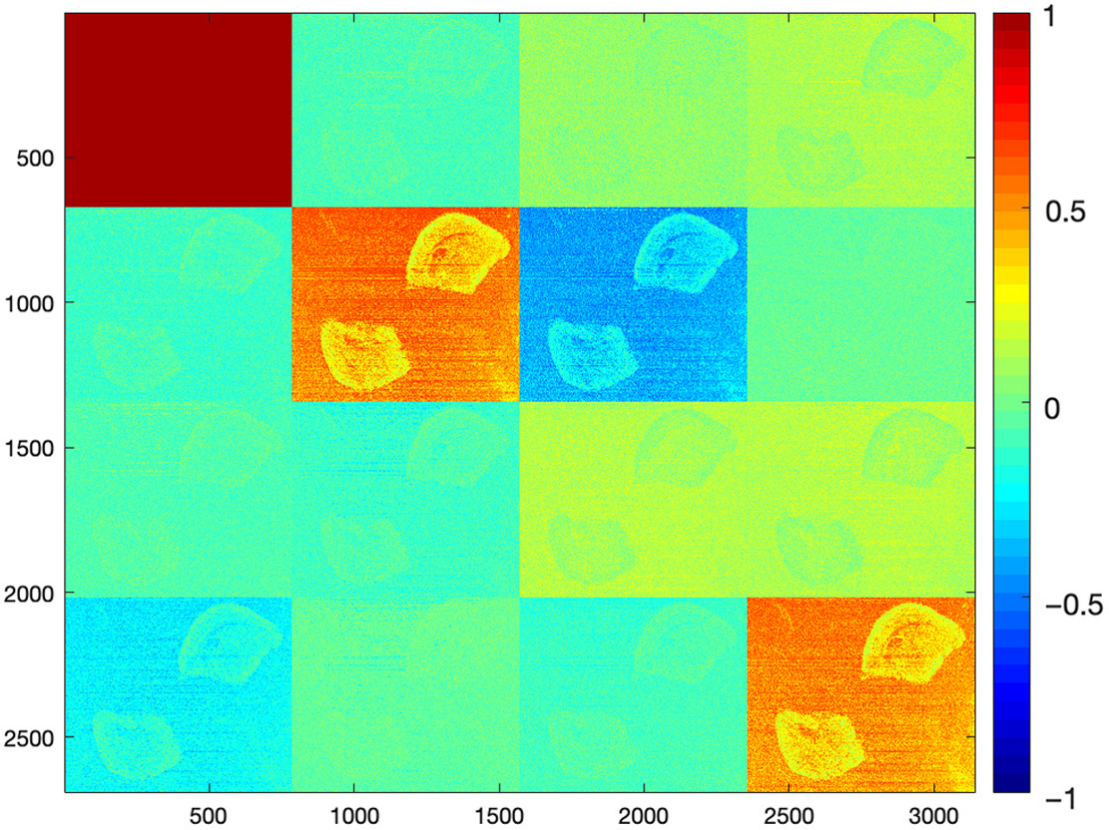
MM example of mouse brain FFPE block (red laser source), age = 225 days.

These qualitative differences provide the basis for the subsequent statistical analysis of selected MM elements and derived depolarization parameters, allowing us to also quantify how sample preparation influences the sensitivity of the polarimetric measurements to Alzheimer Aβ related structural changes (Fig. 5).

### 3.1 Scattering Regimes and Polarimetric Signatures

Based on the brain tissue and signal observed from the diagonal MM elements, the size of the scattering particle (radius a) plays an important role.^25^ Previously published work had reported Rayleigh-like scattering for FFPE block measurements.^17^ With respect to MMP, it is known that for small scatterers, a≪λ, Rayleigh regime is considered where linear polarization has to be preserved better than circular; typically $m_{22}\approx m_{33}>m_{44}$; also the circular polarization tends to depolarize faster because small scatterers randomize light helicity.^26^^–^^28^ For larger scatterers, (a∼λ), Mie regime has to be considered, whereby the circular polarization can be preserved after multiple scattering events; $m_{44}$ becomes comparable to or even larger than $m_{22}$, $m_{33}$; also an increased anisotropy (g) and dominance of forward scattering is expected.

Generally, it is difficult to control and quantify the relevant optical properties in a complex medium such as biological tissue. Experimental depolarization investigations were often carried out using tissue-mimicking phantoms in which the parameters can be precisely defined.^29^ Suspensions of intralipid or aqueous suspensions of polystyrene and silica microspheres are most commonly used.^30^ The size of the scatterers is typically such that the anisotropy is comparable to that of the tissue, whereas the concentration of scatterers is adjusted to yield the desired scattering coefficient $\mu_{s}$ or reduced scattering coefficient $\mu_{s}^{\prime }$.^31^ For media comprised of Rayleigh scatterers, circular polarized light is depolarized more strongly than linear polarization in forward transmission. The overall strength of depolarization is weaker in the Mie regime due to the predominance of forward scattering at high anisotropy values.^32^^,^^33^ As the scatterer size increases, both the scattering distribution and the helicity flip angle change. For Mie scatterers, the helicity flip angle exceeds 90 deg, whereas forward scattering predominates and large-angle events become rare. The result is that helicity is much less frequently randomized, leading to weaker depolarization of circular polarization compared to the Rayleigh case.^34^

Interestingly, it was observed in all the cases that the MM element $m_{44}$ was not suppressed compared with $m_{22}$ and $m_{33}$ but instead reaches comparable or higher values. This behaviour is indicative of a transition away from the Rayleigh regime, where circular polarization is typically lost more rapidly, toward Mie type scattering, where helicity can be preserved through multiple scattering events. The presence of larger scattering centres, such as amyloid plaque aggregates, is therefore consistent with the elevated $m_{44}$ values observed in our measurements. The characteristic of biological tissues undergoing Rayleigh scattering rather than Mie scattering in a backscattering configuration leads to higher $m_{22}$ and $m_{33}$ values compared with $m_{44}$.^16^ At longer wavelengths, the scattering falls within the Rayleigh–Mie transition regime, where the sample acts as an isotropic depolarizer. The case presented in this study is expected to be in the Mie regime as we can assume a larger size of Aβ plaques.

### 3.2 Statistical Analysis

One of the key parameters that previous studies focused on was the analysis of the central moments of the MM values. In the following, the four parameters are given:

1. Mean:
  $$\mu =\frac{1}{N}\overset{N}{\underset{i=1}{\sum }}x_{i},$$
2. Standard deviation:
  $$\sigma =\sqrt{\frac{1}{N}\overset{N}{\underset{i=1}{\sum }}(x_{i}-\mu {)}^{2}},$$
3. Skewness:
  $$\mathrm{Skewness}=\frac{\frac{1}{N}\overset{N}{\underset{i=1}{\sum }}(x_{i}-\mu {)}^{3}}{\sigma^{3}},$$
4. Kurtosis:
  $$\mathrm{Kurtosis}=\frac{\frac{1}{N}\overset{N}{\underset{i=1}{\sum }}(x_{i}-\mu {)}^{4}}{\sigma^{4}},$$
  where $x_{i}$ is the individual data points and N is the number of data points.

**Fig. 6:**
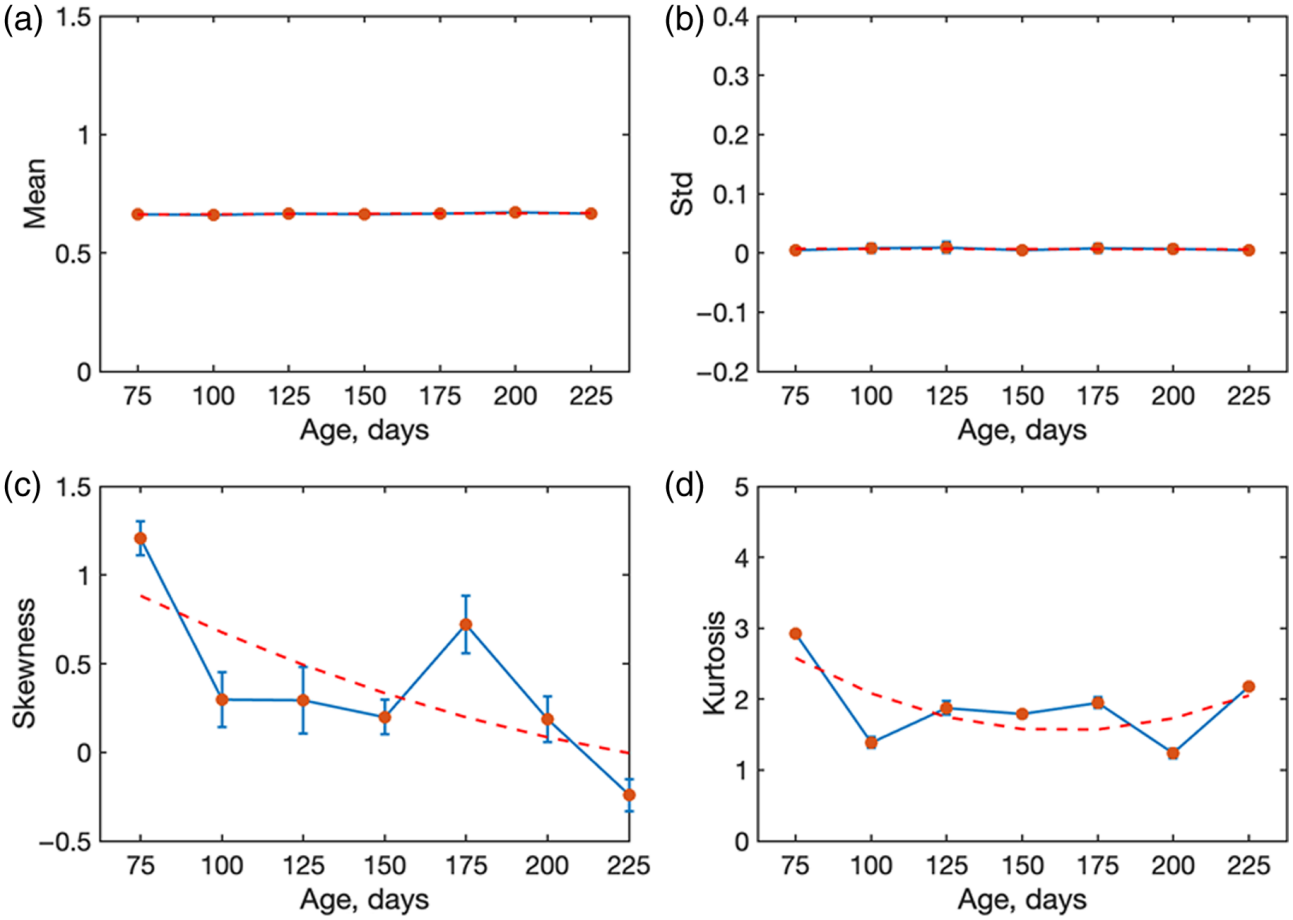
Statistical moments (first to fourth order) describing the spatial distributions of the depolarization measured from mouse brain slides (no paraffin) with AD progression (ages 50 to 225 days) at 630 nm: mean, standard deviation, skewness, and kurtosis. The orange line is the linear fit for panels (a) and (b), and the exponential fit for panels (c) and (d).

For each age group, the statistical moments (first to fourth order) of the MM-derived parameters were calculated and then averaged across the corresponding samples. Our work here extends the statistics analysis beyond $m_{22}$ to $m_{44}$ and decomposition parameters (R, D, P, Δ) and results tested across multiple sample preparations. In Fig. 6, the depolarization results are shown, presenting how the mean, standard deviation, skewness, and kurtosis evolve with animal age. The error bars indicate the variability within each age group. Skewness fluctuates but overall shifts toward positive values with increasing age, indicating distributions becoming more asymmetric, with a tail toward higher depolarization. Kurtosis decreases with age, suggesting that distributions become less peaked and more heavy-tailed as structural disorder increases. It can be said that these higher-order changes reflect microstructural modifications in the tissue (amyloid plaques, aggregation, increased scatterer size). The decreasing kurtosis may indicate more heterogeneous scattering as the disease progresses. In addition, a positive skewness points toward more scattering sites that retain higher depolarization values.

**Fig. 7:**
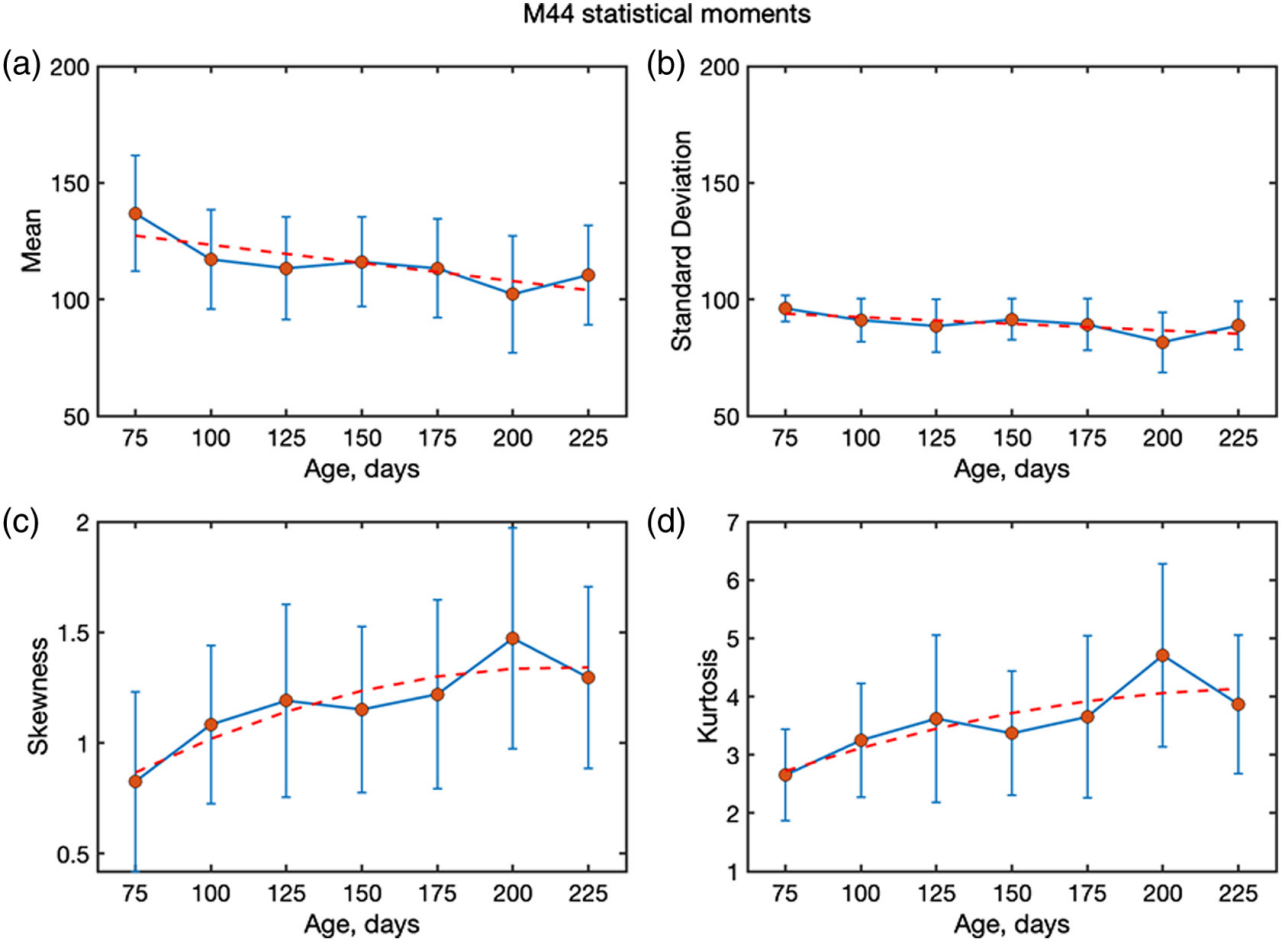
Statistical moments (first to fourth order) describing the spatial distributions of the $m_{22}$ element of the MM measured from mouse brain slides (no paraffin) with AD progression (ages 50 to 225 days) at 630 nm: mean, standard deviation, skewness, and kurtosis. The orange line is the linear fit for panels (a) and (b), and the exponential fit for panels (c) and (d).

A notable aspect is that the values of $m_{22}$ and $m_{44}$ are in good correlation with AD progression. The first and second statistical moments of $m_{22}$ do not show significant changes, whereas higher-order moments exhibit exponential changes with AD progression. The third-order statistical moment of $m_{22}$ [Fig. 7(c)] agrees with an exponential fit, indicating that the distribution is skewed toward higher values. For the fourth-order kurtosis, a similar behavior was observed. Such a difference in higher-order statistics behavior is indicative of heavy-tailed distributions. The structural changes in the AD brain tissue increase the effective scattering cross-section due to the increase in the size of scattering particles. These changes in turn affect the higher-order statistical moments. Thus, these higher-order moments can be a metric that is indicative of AD progression.

**Fig. 8:**
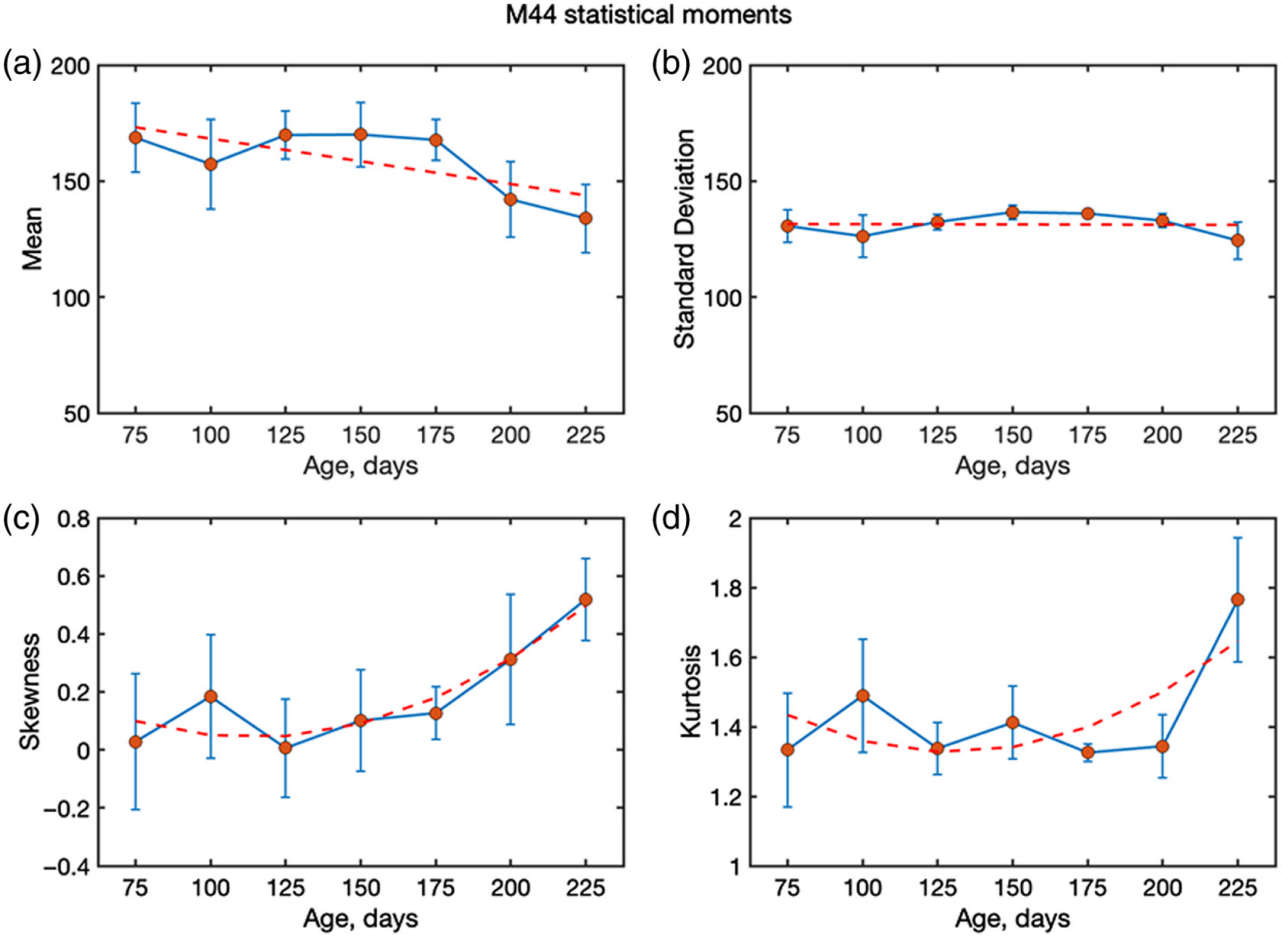
Statistical moments (first to fourth order) describing the spatial distributions of the $m_{44}$ element of the MM measured from mouse brain slides (no paraffin) with AD progression (ages 50 to 225 days) at 630 nm: mean, standard deviation, skewness, and kurtosis. The orange line is the linear fit for panels (a) and (b), and the exponential fit for panels (c) and (d).

The observation of high $m_{44}$, as discussed earlier, indicates a shift toward Mie scattering. An important observation from the measurements is the unusually high contribution of the $m_{44}$ element compared with earlier reports on FFPE blocks,^16^ where $m_{44}$ was typically lower than $m_{22}$ and $m_{33}$. In the presented work, $m_{44}$ reaches comparable or even higher values, indicating that circular polarized light is preserved more strongly than expected. This behavior is consistent with Mie type scattering, where helicity preservation is enhanced due to the presence of larger scattering locations such as amyloid aggregates. To quantify this effect, the statistical moments (first to fourth order) of $m_{44}$ as a function of age were analyzed. Figure 8 shows the mean, standard deviation, skewness, and kurtosis of the $m_{44}$ distributions across all age groups. Although the mean and standard deviation decrease slightly or even remain constant with age, skewness and kurtosis both increase, particularly beyond an age of 175 days, suggesting that the underlying distributions become more asymmetric and heavy-tailed as the disease progresses. These trends reinforce the idea that $m_{44}$ provides additional sensitivity to structural changes in brain tissue and highlight its potential as a marker of AD-related scattering alterations. The insensitivity of $m_{22}$ and the total depolarization moments can be explained by regional variability and the compensating effects of scattering anisotropy. However, $m_{44}$ behaves differently, reflecting the stronger survival of circularly polarized light in Mie-dominated regimes. Its age-dependent trends therefore provide evidence that the tissue scattering regime shifts with AD progression, from predominantly Rayleigh behaviour toward conditions where Mie scattering dominates. This makes $m_{44}$ skewness/kurtosis a new, plaque-sensitive marker of AD progression.

**Fig. 9:**
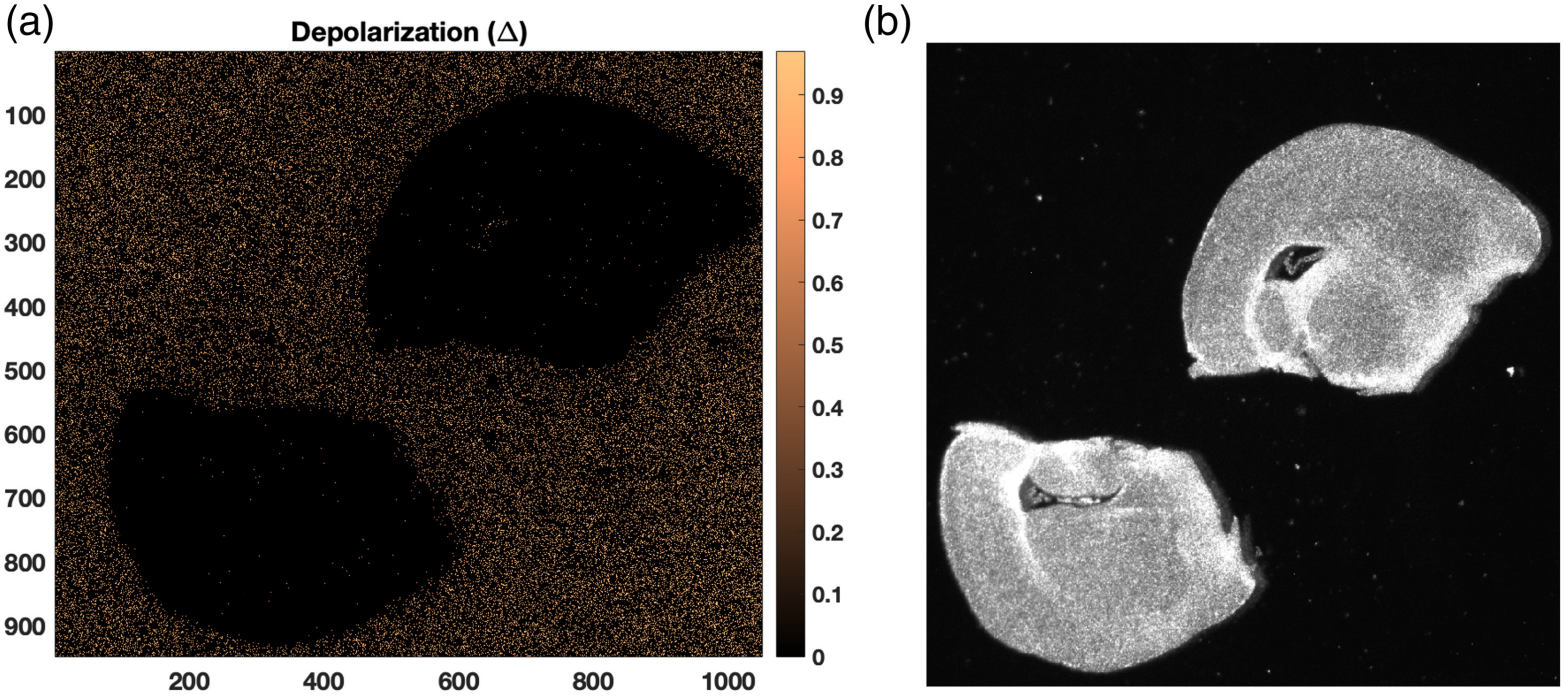
(a) Depolarization mapping of the brain slides. (b) Image of brain tissue slices of 225-day-old mouse without paraffin. Low depolarizing power is observed in the range of 0 to 0.2.

To visualize the spatial distribution of depolarizing properties across the tissue, Fig. 9 presents a representative depolarization (Δ) image of a mouse brain section alongside the corresponding bright-field image. The depolarization map highlights regions where the incident polarization state is strongly changed, shown in the warmer color scale. By contrast, regions of low depolarization (dark values) correspond to tissue structures that preserve polarization more effectively. As can be seen, the overall depolarization is low within the brain section compared with the surrounding medium, consistent with the anisotropic scattering properties of tissue. Local variations across different anatomical regions are clearly visible, providing a spatially resolved contrast that complements the statistical analysis of the MM elements. Such maps can therefore serve as an intuitive tool for linking polarimetric metrics with tissue morphology and disease progression. Depolarization is also strongly influenced by the refractive index contrast between scatterers and the surrounding medium. The depolarization characteristics of such low contrast Mie media resemble those of Rayleigh scatterers.^34^^,^^35^ Therefore, its depolarization behavior is expected to resemble that of Rayleigh-type media. This has been consistently observed in experimental studies on tissue, despite the commonly cited paradigm that circular polarization is better preserved in Mie scattering. This counter-intuitive behavior occurs because low refractive index scatterers with moderate to large sizes still operate within the weak scattering regime. Each volume element within the particle can be approximated as producing independent dipole-like scattering, making the scattering matrix effectively Rayleigh like. As a result, such weakly fluctuating anisotropic media exhibit depolarization characteristics similar to Rayleigh scatterers. Biological tissue itself can be approximated as a random continuum of inhomogeneities with weak refractive index fluctuations. However, it had to be noted that in anisotropic media, circularly polarized photons detected in the backward direction may arise either from helicity-flipped backscattering events or from helicity-preserving photons that undergo multiple forward scattering before emerging backward.

From the Mueller matrix decomposition, a set of characteristic polarimetric parameters can be derived, each describing a specific aspect of the sample’s optical behavior.^36^ In addition to $m_{22}$ and $m_{44}$, we evaluated the statistical behavior of the decomposition parameters: retardance (R), polarizance (P), diattenuation (D), and depolarization (Δ). Table 2 lists the mean, standard deviation, skewness, and kurtosis values of these parameters for all investigated age groups. Recent work on isotropic depolarization filtering in bioimaging suggests that selectively removing the isotropic component of Δ can uncover additional structural contrast in polarimetric images.^37^ Applying such filters to MMP data from AD brain tissue could further emphasize anisotropic scattering associated with amyloid plaques and provide an even richer feature space for quantitative analysis and machine learning models and thus represents a promising direction for follow-up studies. To our knowledge, previous AD-related MMP studies did not report decomposition parameters (R,D,P, Δ) or their higher-order moments, making these parameters a new potential polarimetric window into microstructural changes beyond the diagonal MM elements.

**Table 2:** Statistical moments of polarimetric parameters across sample age. Ages in days. Samples are brain slides without paraffin and illuminated by a red laser source at 632 nm.

	75	100	125	150	175	200	225
R							
Mean	0.665	0.627	0.650	0.624	0.636	0.665	0.618
Std	0.019	0.012	0.007	0.006	0.003	0.003	0.002
Skew	−1.018	0.695	−0.771	1.078	1.103	0.726	0.928
Kurt	3.509	1.620	2.671	2.970	2.292	2.203	3.160
P							
Mean	0.252	0.229	0.250	0.230	0.242	0.258	0.219
Std	0.015	0.017	0.005	0.005	0.003	0.001	0.003
Skew	−1.415	0.658	−0.650	0.176	1.052	−0.092	0.734
Kurt	3.437	1.513	3.094	1.988	2.242	1.686	2.477
D							
Mean	0.109	0.166	0.113	0.141	0.129	0.107	0.165
Std	0.031	0.032	0.008	0.007	0.001	0.003	0.018
Skew	1.591	−0.676	1.164	−1.173	0.002	−0.708	0.275
Kurt	4.367	1.505	3.073	3.082	1.098	2.162	1.616
Depol.							
Mean	0.505	0.436	0.474	0.438	0.451	0.498	0.430
Std	0.048	0.018	0.011	0.006	0.004	0.003	0.004
Skew	0.699	0.728	−1.432	1.397	1.096	−0.622	0.497

The results show that the mean values of retardance and depolarization remain relatively stable across ages, whereas diattenuation exhibits larger variability. Higher-order moments highlight additional contrasts: for instance, skewness of retardance shifts from negative at early ages to positive at later ages, suggesting changes in the distribution symmetry with disease progression. Similarly, kurtosis values fluctuate with age, reflecting increased heterogeneity of tissue scattering. These complementary metrics provide additional polarimetric markers beyond the diagonal elements of the MM, further supporting the sensitivity of polarization analysis to AD’s structural alterations.

The exponential changes observed in the higher-order statistical moments provide important insights into the scattering regime of AD-affected tissue. An increase in the third moment (skewness) indicates that the distributions of depolarization become increasingly asymmetric with disease progression, whereas the growth of the fourth moment (kurtosis) reflects the emergence of heavy-tailed distributions with stronger contributions from outliers. These behaviors arise from structural malformations in AD brain tissue, where the accumulation of amyloid plaques increases the effective scatterer size and enhances the scattering cross-section. As a consequence, light scattering shifts toward a more anisotropic, Mie-like regime with stronger angular dependence of the scattering phase function. The growing spatial inhomogeneity of the tissue further amplifies the irregularity of photon trajectories, leading to greater variability in the degree of polarization retained by photons exiting the tissue. This explains the higher absolute values of skewness and kurtosis observed in our data.

Importantly, in our case, not only linear depolarization ($m_{22}$) but also the circular channel ($m_{44}$) exhibits age-dependent changes, with skewness and kurtosis of $m_{44}$ increasing systematically at later stages. This suggests that circular polarization is particularly sensitive to the enlarged and heterogeneous scattering centres characteristic of AD pathology. Taken together, these findings highlight that higher-order statistical moments of both linear and circular depolarization provide more sensitive metrics for monitoring disease progression than the mean or standard deviation alone.

### 3.3 Physical Interpretation

The quantification of the distribution of polarimetric parameters (e.g., $m_{22}$, $m_{44}$, depolarization, or anisotropy) for biological samples can yield insights into the physical interpretation of the first four central statistical moments: mean, variance, skewness, and kurtosis. Here, we summarize the physical and optical content of each moment in the specific context of local scattering distribution, microstructural heterogeneity, scatterer size and anisotropy, helicity preservation, and plaque-driven refractive index fluctuations.

The first-order moment (mean, μ) captures the average value of a given polarimetric observable over the field of view. In terms of local scattering distribution, a higher μ corresponds to a higher typical probability that a photon experiences helicity randomizing interactions before exiting the tissue. It has been established that the mean depolarization (Δ) increases systematically with the scattering coefficient $\mu_{s}$ in phantoms and is higher in cancerous versus healthy paraffin-embedded colorectal tissue, reflecting a higher overall multiplicity of scattering and structural disorder.^38^ For AD plaques, an increase in $m_{44}$ or in a mean circular polarization-related metric at later ages can be interpreted as a shift in the baseline scattering regime, driven by an increased average refractive index contrast or by the accumulation of higher-order Mie scatterers associated with compact plaques.

For local scattering distributions, a large variance ($\sigma^{2}$) indicates substantial spatial variability in the local scattering regime. The variance of reconstructed differential anisotropy parameters that discriminates between fibrillar and polycrystalline tissue architectures has been demonstrated.^39^ Physically, a high σ points toward greater microstructural heterogeneity, i.e., a more heterogeneous mixture of regions with preserved neural fibre alignment and relatively low refractive index contrast (near normal scattering) and regions with strongly perturbed structure. It has been reported that microscopic heterogeneity (mixtures of structural compartments) can strongly change depolarization, even if bulk $\mu_{s}$ and g are matched.

The third-order moment, skewness, in a depolarization-related quantity means that most regions are weak or moderate scatterers, but a minority of pixels exhibit extremely strong scattering or anisotropy. Skewness of Mueller matrix elements is one of the most sensitive diagnostically relevant parameters for distinguishing structured (e.g., polycrystalline collagen) from parenchymal tissues and for detecting tissues in which cancer disrupts this behaviour in the intermediate size regime, effectively corresponding to a change in the shape and asymmetry of the underlying distribution.^38^^,^^39^ A positive skewness of $m_{44}$ or of a helicity-related metric can be interpreted as evidence for rare but strong helicity-altering domains embedded in a more weakly polarimetric background. AD plaque-rich regions, with pronounced refractive index contrast and size parameters in the Mie regime, are expected to act as such “extreme” domains, shifting the tail of the distribution.

The fourth-order moment, kurtosis, has been shown to respond strongly to changes in the heterogeneity and organization of the polycrystalline network, similarly to skewness. The diagnostic importance of heavy-tailed distributions has been demonstrated in detecting pathological changes in the polycrystalline structure of tissues, with two- to three-fold differences between tissue types.^38^ For our AD model, elevated kurtosis of a plaque-sensitive metric (e.g., $m_{44}$ or depolarization) indicates that extreme scattering or anisotropy events are not only present but spatially concentrated in plaque-rich regions.

The above interpretation of the central moments is consistent with the literature, which shows that in polydisperse media even a small volume fraction of subwavelength scatterers can drastically reduce helicity preservation, and that multiple scattering in forward direction (Mie like) versus isotropic (Rayleigh like) environments leads to distinct trajectories of the Stokes vector and different rates of depolarization for linear and circular states.^40^^,^^41^ Our interpretation bridges spatial polarimetric imaging, microstructural heterogeneity, and single scatterer polarization memory and provides insights into how the observed statistical trends may relate to the underlying physics of scattering and to plaque-driven structural remodelling in AD brain tissue.

### 3.4 AD Brain Tissue with Paraffin

**Fig. 10:**
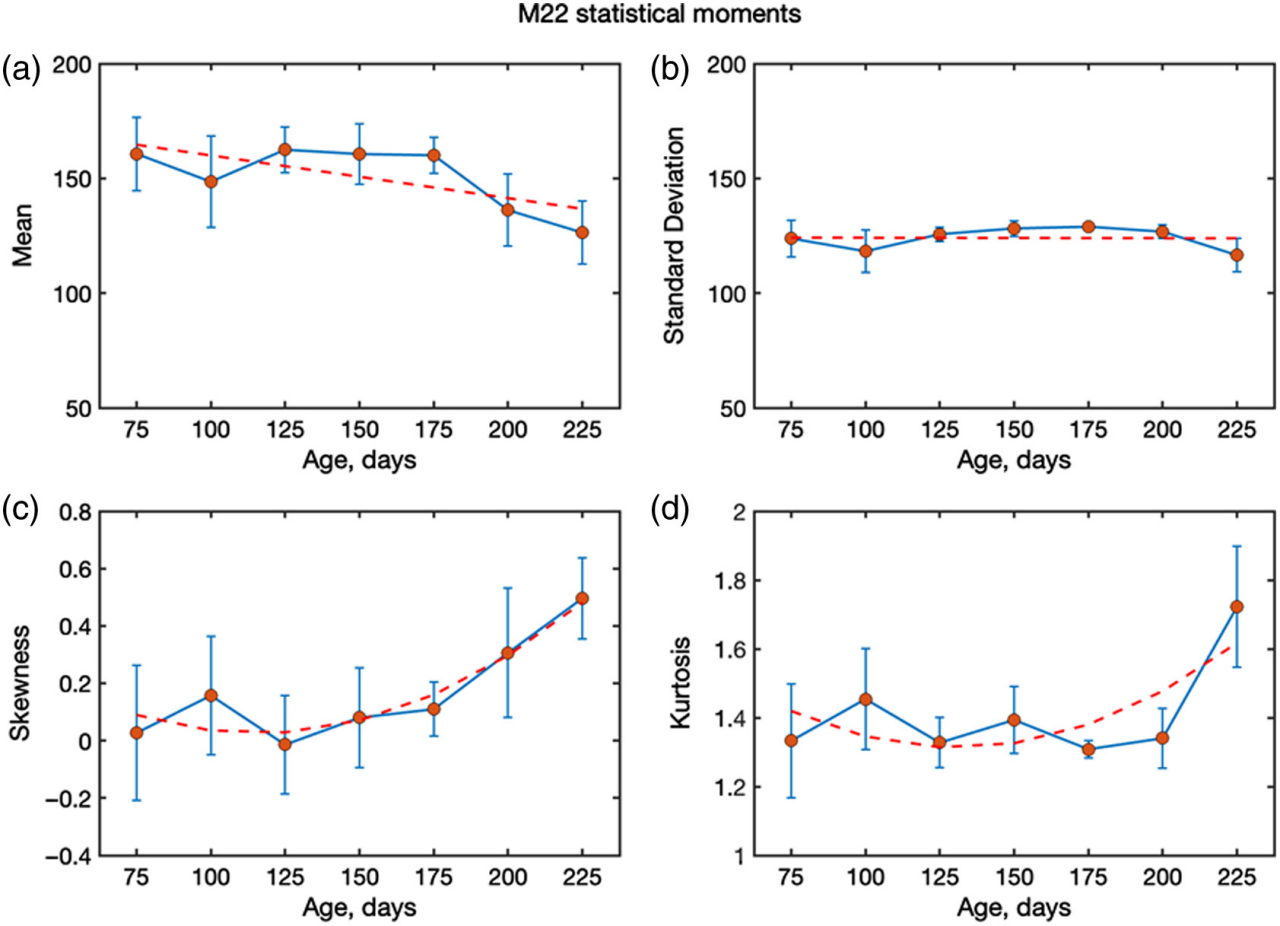
Statistical moments (first to fourth order) describing the spatial distributions of the $m_{44}$ measured from mouse brain slides (with paraffin) with AD progression (ages 50 to 225 days) at 445 nm: mean, standard deviation, skewness, and kurtosis. The orange line is the linear fit for panels (a) and (b), and the exponential fit for panels (c) and (d).

**Fig. 11:**
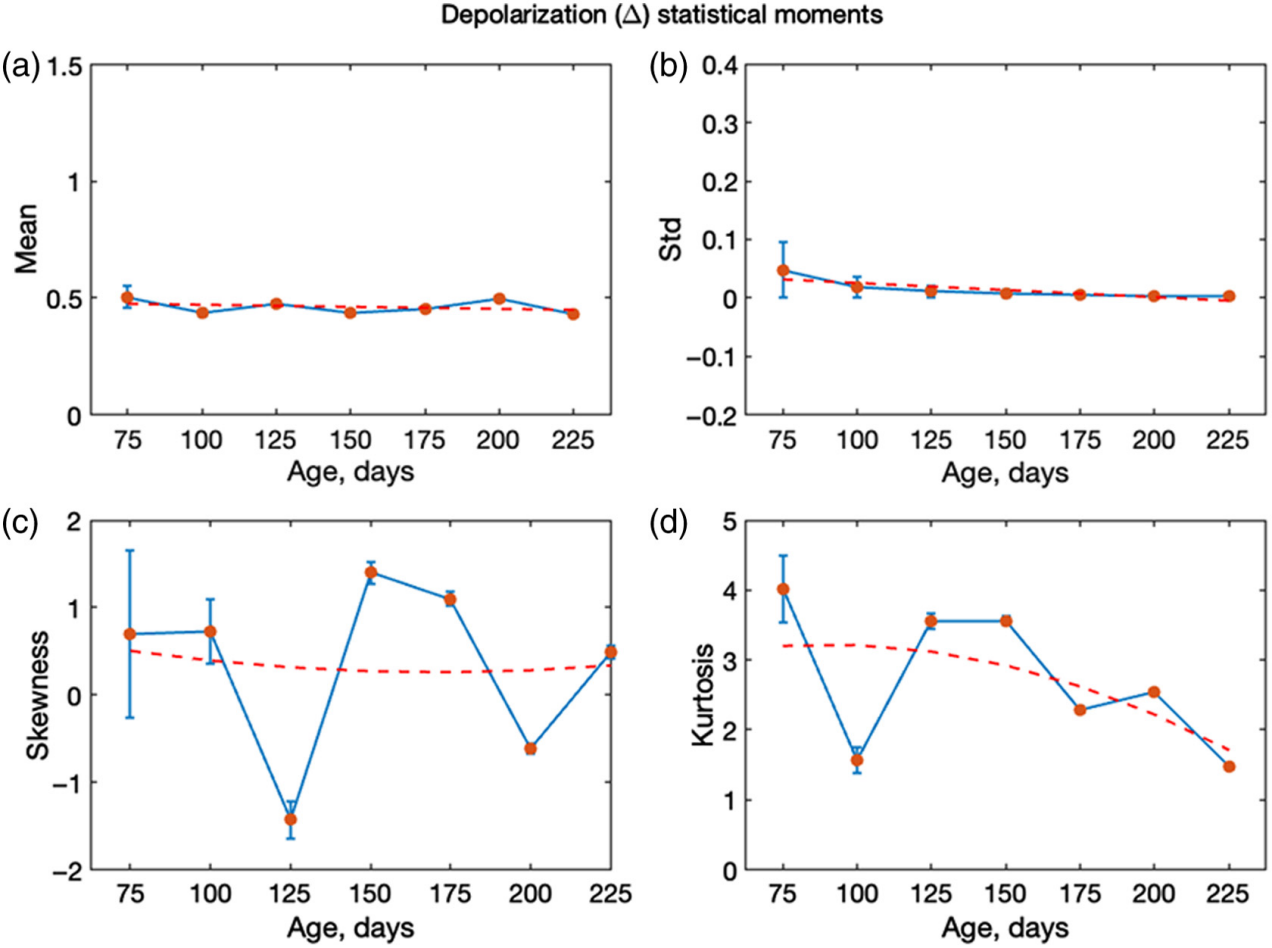
Statistical moments (first to fourth order) describing the spatial distributions of the depolarization measured from mouse brain slides (with paraffin) with AD progression (ages 50 to 225 days) at 445 nm: mean, standard deviation, skewness, and kurtosis. The orange line is the linear fit for panels (a) and (b), and the exponential fit for panels (c) and (d).

As described in Sec. 2.1, a set of measurements was also performed on tissue slides embedded in paraffin. The overall behavior of the statistical moments follows the same age-dependent trends as the ones for the samples without paraffin (Figs. 10 and 11). However, the presence of paraffin reduces the absolute values of both $m_{22}$ and $m_{44}$, consistent with the additional depolarizing influence of the embedding medium. Despite this reduction in magnitude, the progression with age remains visible. In particular, skewness and kurtosis continue to provide useful markers, although their contrast is diminished compared with the nonparaffin case. This suggests that although paraffin embedding attenuates polarimetric sensitivity, higher-order statistics can still capture microstructural changes associated with AD progression. An important new practical diagnostic insight, compared with the previous work, is the emergence of $m_{44}$ as a robust marker of AD progression. Across all sample preparations investigated (paraffin-free sections, paraffin-embedded sections, and FFPE blocks), the present results consistently highlight the diagnostic relevance of $m_{44}$, underscoring its potential as a preparation-insensitive polarimetric biomarker.

Practically, our findings are most directly translatable as an adjunct to standard neuropathology on FFPE and paraffin sections, where polarimetric imaging could provide quantitative, label-free metrics of plaque burden and microstructural heterogeneity within existing slide-scanning workflows. In the longer term, the same polarimetric contrast mechanisms and analysis framework may inform the design of *in vivo* or minimally invasive approaches, for example, retinal polarimetric imaging as an indirect window into AD-related tissue changes, although such applications will require dedicated clinical validation.

## 4 Conclusion

In this study, we employed MMP to investigate structural and compositional alterations in brain tissues across different age groups and preparation conditions. By analyzing both paraffin-embedded and nonparaffin samples, we found that age-dependent trends are present in both sample sets that although embedding reduces the absolute magnitude of polarimetric parameters such as $m_{22}$ and $m_{44}$. Importantly, higher-order statistics, including skewness and kurtosis, provided additional sensitivity to subtle microstructural variations, offering a richer description of tissue heterogeneity than mean values alone. The results indicate that MMP, combined with statistical moment analysis, can serve as a powerful label-free approach for quantifying disease progression and tissue alterations in neurodegenerative models. These findings highlight the potential of polarimetric techniques to complement conventional histology by offering rapid, objective, and reproducible characterization of brain tissue microstructure. Future work will focus on extending the methodology to three-dimensional reconstructions, correlating polarimetric signatures with biochemical markers, and validating the approach on larger cohorts to strengthen its translational relevance. The entire dataset presented here provides a substantially richer feature space for future machine learning approaches. This extended representation is particularly advantageous for data-driven classification and for investigating domain adaptation between different histological preparations, thereby facilitating eventual clinical translation. The polarimetric measurement of isolated Aβ peptides would deepen our fundamental understanding of their optical response and provide a valuable reference measurement.

## Data Availability

The data associated with this paper are not public but can be made available upon reasonable request.
